# Genome-Wide DNA Methylation Comparison between *Brassica napus* Genic Male Sterile Line and Restorer Line

**DOI:** 10.3390/ijms19092689

**Published:** 2018-09-10

**Authors:** Zhixin Wang, Xiangping Wu, Zengxiang Wu, Hong An, Bin Yi, Jing Wen, Chaozhi Ma, Jinxiong Shen, Tingdong Fu, Jinxing Tu

**Affiliations:** 1 National Key Laboratory of Crop Genetic Improvement, College of Plant Science and Technology, National Sub-Center of Rapeseed Improvement in Wuhan, Huazhong Agricultural University, Wuhan 430070, China; watc1989@163.com (Z.W.); wuxiangp@webmail.hzau.edu.cn (X.W.); wuzengx@webmail.hzau.edu.cn (Z.W.); yibin@mail.hzau.edu.cn (B.Y.); wenjing@mail.hzau.edu.cn (J.W.); yuanbeauty@mail.hzau.edu.cn (C.M.); jxshen@mail.hzau.edu.cn (J.S.); futing@mail.hzau.edu.cn (T.F.); 2 Division of Biological Sciences, University of Missouri, Columbia, MO 65211, USA; anho@missouri.edu

**Keywords:** *Brassica napus*, genic male sterility, floral bud, whole-genome bisulfite sequencing

## Abstract

DNA methylation is an essential epigenetic modification that dynamically regulates gene expression during plant development. However, few studies have determined the DNA methylation profiles of male-sterile rapeseed. Here, we conducted a global comparison of DNA methylation patterns between the rapeseed genic male sterile line 7365A and its near-isogenic fertile line 7365B by whole-genome bisulfite sequencing (WGBS). Profiling of the genome-wide DNA methylation showed that the methylation level in floral buds was lower than that in leaves and roots. Besides, a total of 410 differentially methylated region-associated genes (DMGs) were identified in 7365A relative to 7365B. Traditional bisulfite sequencing polymerase chain reaction (PCR) was performed to validate the WGBS data. Eleven DMGs were found to be involved in anther and pollen development, which were analyzed by quantitative PCR. In particular, *Bnams4* was hypo-methylated in 7365A, and its expression was up-regulated, which might affect other DMGs and thus control the male sterility. This study provided genome-wide DNA methylation profiles of floral buds and important clues for revealing the molecular mechanism of genic male sterility in rapeseed.

## 1. Introduction

Oil rapeseed (*Brassica napus* L.; A_n_A_n_C_n_C_n_) is one of the most important sources of edible vegetable oils in the world. *B. napus* was derived from spontaneous allopolyploidization between the ancestors of *Brassica oleracea* (C_o_C_o_) and *Brassica rapa* (A_r_A_r_) about 7500 years ago [1]. In spite of the common ancestor, *B. oleracea* and *B. rapa* respectively contain 38.80% and 21.47% transposable elements (TEs) in the assembled genomes [2]. The above facts lead to the asymmetric distribution of TEs in the A_n_ and C_n_ subgenomes of assembled *B. napus* genome, which may lead to different methylation patterns between the two subgenomes [1].

Male sterility (MS) is important in crop breeding because it greatly facilitates the production of hybrid seeds and utilization of heterosis on a large scale [3]. MS exists widely in flowering plants since male reproductive development can be disturbed in various processes, including stamen specification, anther cell division or differentiation, microsporocyte meiosis, tetrad microspore release, microspore mitosis, pollen wall development, and anther dehiscence [4]. Numerous genes constitute a complex regulatory network of anther and pollen development [5]. Developmental reprogramming of DNA methylation can affect gene expression during plant sexual reproduction, and the RNA-directed DNA methylation (RdDM) pathway potentially regulates the development of many cell types and tissues [6]. Therefore, many studies have focused on DNA methylation in male reproductive development [7,8].

DNA methylation, an important epigenetic modification process in which methyl groups are transferred to the cytosines of DNA, can regulate gene expression without altering the DNA sequences. Plant DNA methylation occurs in all different sequence contexts including CG, CHG, and CHH (H represents either A, T, or G) [9]. DNA methylation is essential for normal plant development and is associated with several key processes including regulating gene expression, silencing/activating transposon, remodeling chromatin, and maintaining genome stability [10,11]. Generally, DNA methylation is inversely correlated with gene expression. However, the function of DNA methylation varies with the genomic targets [12]. The DNA methylation of regulatory elements such as promoters and enhancers potentially down-regulates gene expression by altering the chromatin structure and blocking transcription initiation. The DNA methylation of repeat sequences is also linked to silencing effects. Gene body methylation is not necessarily associated with the repression of gene expression, and instead exhibits extensive positive correlations with transcription activation. Besides, it may also play an important role in silencing repetitive elements and alternative splicing [12,13]. DNA methylation is subject to dynamic regulation in the response to developmental and environmental cues.

In plants, the methylation levels and patterns vary across species, tissues or organs, developmental status and the biotic and abiotic environment [14]. Whole-genome bisulfite sequencing (WGBS) has emerged as a powerful approach for studying DNA methylation by providing genome-wide methylation profiles at single-base resolution [15]. Recently, WGBS has been widely applied to profiling the DNA methylation in many plants, such as *Arabidopsis* [16], rice [17], soybean [18], tomato [19], cotton [20] and cabbage [21]. It was also used to investigate the roles of DNA methylation in plant MS, especially since Ding et al. found that *Psi–LDMAR* reduced the expression of *PMS3* through RdDM to regulate photoperiod-sensitive male sterility in rice [22]. Li et al. identified eight key differentially methylated genes that are possibly associated with cytoplasmic male-sterility (CMS) in soybean [23]. By comparing the DNA methylome of cabbage genic male sterile (GMS) 01-20S and its fertile line, Han et al. identified nine genes related to pollen development, among which *Bol039180* might be involved in pectin degradation and pollen separation [21].

The rapeseed GMS line 9012A is a spontaneous mutant found in 1992 [24]. The genes governing male sterility in 7365A were revealed to be allelic to those in 9012A [25]. The 7365ABC three-line pollination control system provides an effective breeding strategy for the commercial rapeseed hybrids. In addition to the advantages such as stable sterility, wide distribution of restorer lines and almost no negative cytoplasmic effects, this system can produce a completely male sterile line by cross pollination between male sterile and temporary maintainer lines [26,27].

The male fertility of rapeseed 7365ABC is controlled by two independent loci: *Bnams4^a^/Bnams4^b^/Bnams4^c^*, a multiallelic locus on chromosome A07; and *BnaMs3/Bnams3*, which is located on chromosome C09. *BnaMs3* has been characterized as a gain-of-function gene, and is required for tapetal development and function in lipid accumulation in tapetal plastids [28,29]. *Bnams4^b^* is a chimeric gene that is deleterious to tapetal plastids and leads to anther abortion. *Bnams4^a^*, a suppressor of *Bnams4^b^*, was speculated to block the transcription of *Bnams4^b^* by DNA methylation [30]. Several studies have been implemented to reveal the mechanism of this pollination control system [31,32]. Cytological, transcriptional and proteomic comparisons have revealed that 7365A mutant is defective in callose degradation, pollen-wall formation and the transition of the tapetum to the secretory type [31,32,33]. However, the roles of DNA methylation in the regulatory mechanism of rapeseed male sterility remain to be elucidated.

To investigate the profile of DNA methylation and possible function of cytosine methylation in rapeseed male sterility, we performed whole-genome bisulfite sequencing (WGBS) of the floral buds in the male sterile line 7365A and its corresponding fertile line 7365B. The genome-wide DNA methylation in the floral buds of rapeseed was analyzed. Significantly lower methylation was observed for the A_n_ subgenome than for the C_n_ subgenome. Besides, a number of differentially methylated regions (DMRs) and genes related to pollen development were identified. In particular, *Bnams4* was found to be hypo-methylated in the gene body, and its expression was up-regulated in the male sterile line. Our results provide significant insights into the genome-wide DNA methylation profiles of floral buds and important information for further studying the molecular mechanism of genic male sterility in rapeseed.

## 2. Results

### 2.1. Phenotypic Observation of 7365A and 7365B

**Figure 1:**
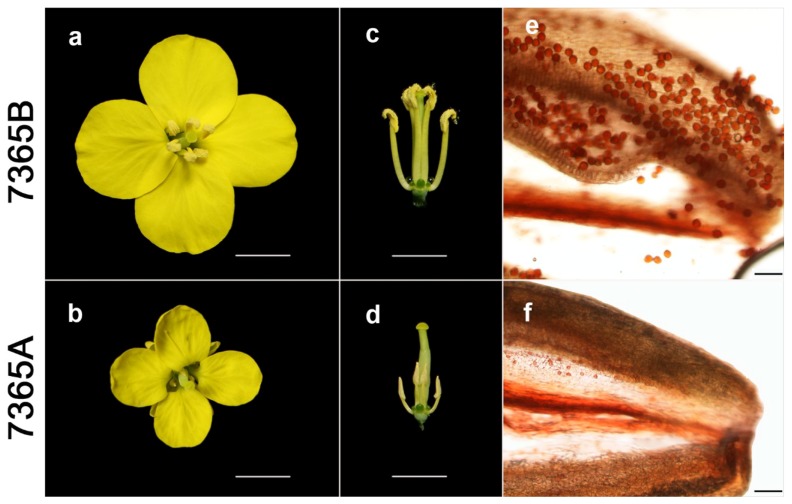
Phenotypic characterization of fertile and sterile lines. (**a**,**b**) Vertical-view of open flowers; (**c**,**d**) Side-view of flowers without calyxes and petals; (**e**,**f**) Acetocarmine-stained anthers; a, c and e are 7365B; b, d and f are 7365A. The scale bars are 5 mm for a, b, c and d, and 100 μm for e and f.

The lines 7365A and 7365B displayed similar normal vegetative growth as well as female fertility, as indicated by the generation of full seeds under open pollination in the field for both lines. However, compared with 7365B, 7365A had smaller petals in the open flower (Figure 1a,b); unlike wild-type anthers with plenty of pollen, the anthers of 7365A were wizened without visible pollen grains (Figure 1c,d). The pistils of the two lines showed almost the same length, but the stamen length of 7365A was approximately half that of 7365B (Figure 1c,d). Furthermore, acetocarmine staining revealed that the 7365B anthers produced many viable round pollen grains, which were released from the anthers under gentle pressing and dyed in deep red. In contrast, the 7365A anthers contained a few lightly-dyed small pollen grains (Figure 1e,f). In brief, 7365A was completely male sterile.

### 2.2. Profiles of Genome-Wide DNA Methylation in 7365A and 7365B

To understand the possible function of cytosine methylation in rapeseed male sterility, young floral buds from male-sterile 7365A and male-fertile 7365B were collected for the construction of bisulfite-treated genomic DNA libraries. In total, whole-genome bisulfite sequencing (WGBS) generated 230 million and 228 million raw reads from the two libraries by paired-end sequencing with Hiseq2500, respectively. Of the 225 million clean reads from the 7365B library, 69.41% (156 million) were uniquely mapped to the rapeseed reference genome of “*Darmor-bzh*” (http://www.genoscope.cns.fr/brassicanapus/data/), while of the 223 million clean reads from the 7365A library, 67.79% (151 million) were uniquely mapped to the rapeseed genome, exhibiting an average read depth of 41.63 and 40.15, respectively. More than 72% cytosines were covered by at least five reads in the rapeseed genome. The depth and density of the sequencing were sufficient for a high-quality genome-wide methylation analysis. Meanwhile, the bisulfite conversion efficiencies represented by the lambda DNA added into the libraries were over 99%, providing reliable and accurate results for the WGBS (Table 1).

**Figure 2:**
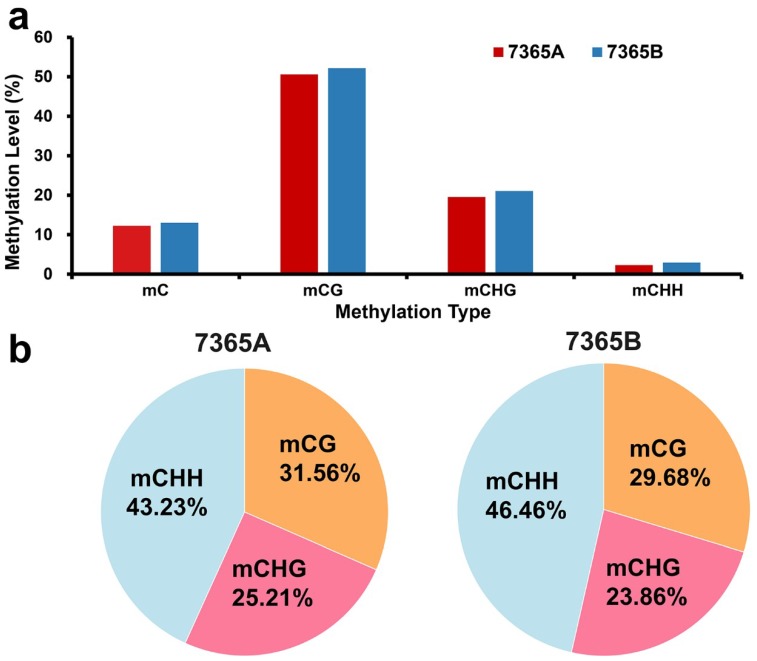
Genome-wide DNA methylation patterns of 7365A and 7365B. (**a**) Average methylation levels of CG, CHG and CHH in the whole genome; (**b**) Percentages of methylated cytosines under each context.

As shown in Figure 2a, 7365B and 7365A showed no significant differences in average methylation levels in the whole genome and under each context. The genome-wide cytosine methylation levels under CG, CHG and CHH contexts were calculated. It is worth noting that among all the sequence contexts, CG had the highest methylation density, followed by CHG and CHH. Although a very low percentage of CHH was methylated, methylated cytosines in the CHH context accounted for a relatively large proportion of the total methylated cytosines (Figure 2b).

**Figure 3:**
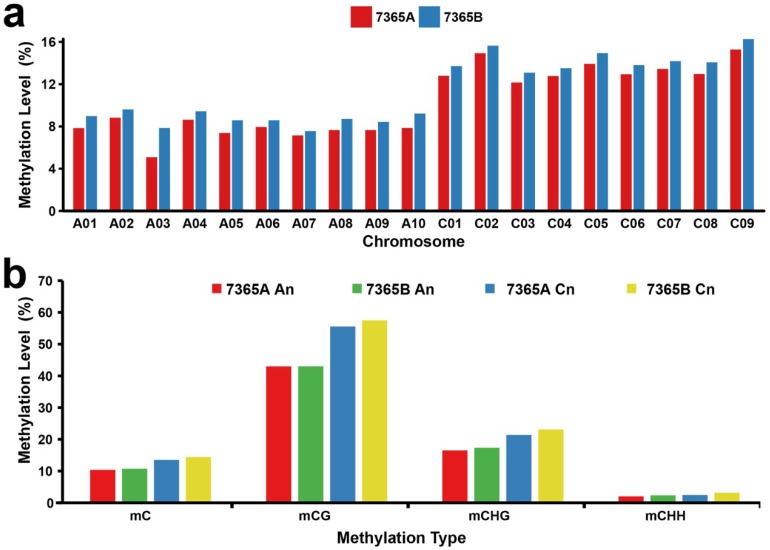
Chromosomal distribution of the global DNA methylation patterns of 7365A and 7365B. (**a**) DNA methylation levels in nineteen chromosomes. (**b**) Mean DNA methylation levels of the A_n_ and C_n_ subgenomes.

To present the global DNA methylation profiles of the two lines, we further analyzed the DNA methylation levels throughout the nineteen chromosomes in rapeseed floral buds under each context. The results showed that DNA methylation was unevenly distributed on the chromosomes (Figure 3a,b).

As the methylation level of the C01 to C09 chromosomes seemed to be obviously higher than that of the A01 to A10 chromosomes in both lines, we separately calculated the mean methylation levels of the A_n_ and C_n_ subgenomes. The Mann–Whitney-U-test showed that the mean methylation level of the A_n_ subgenome was significantly lower than that of the C_n_ subgenome under the CG, CHG and CHH contexts in both lines with *p*-value < 0.01 (Figure 3 and Figure S2).

**Figure 4:**
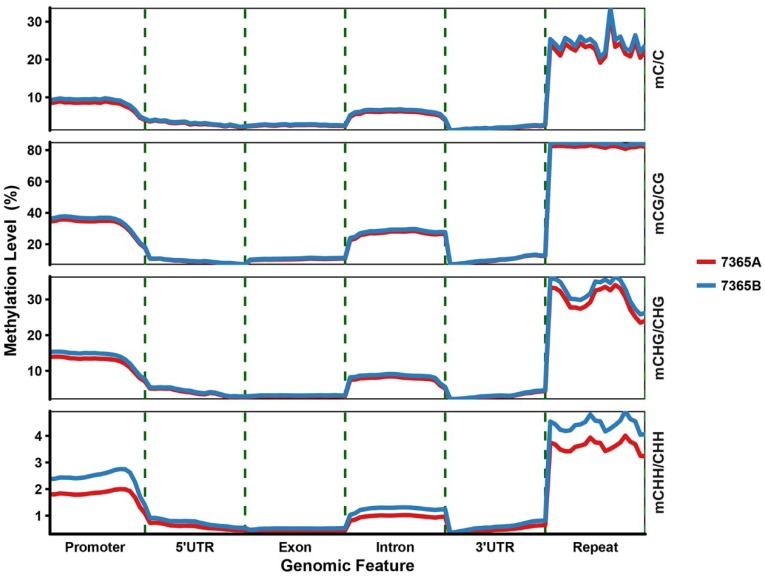
Average density of DNA methylation in different genomic functional regions in 7365A and 7365B. Each functional region was equally divided into 20 bins, and the mean density of the methylated cytosines was defined as the methylation density in each bin.

To better understand the relationship between DNA methylation and gene expression, the genome was divided into several functional regions, namely promoter (2 kb region upstream of transcription start sites, TSSs), gene body (5′untranslated region, (UTR), exons, introns and 3′UTR), and repeat (transposons and other repetitive elements). We observed that the variation tendency of DNA methylation across different genomic regions was consistent between the two libraries under all methylation contexts (Figure 4 and Figure S6). The methylation level was the highest for the repeat compared with other methylated genomic regions, and that of the promoter greatly increased beyond the TSSs. The lowest methylation level was observed in the gene body, with a slightly higher methylation level for introns than for exons.

### 2.3. DNA Methylation Patterns of Genes

Although no significant changes were observed in the genome-wide methylation, there were several local differences (Figure S1). The differentially methylated regions (DMRs) between the two lines were profiled. In total, 779 DMRs were detected, including 136 hypermethylated and 643 hypomethylated DMRs in 7365A relative to 7365B (Table S2). We noticed that there were obviously more hypomethylated DMRs, implying that DNA hypomethylation may occur at the initial stage of anther abortion in 7365A.

**Figure 5:**
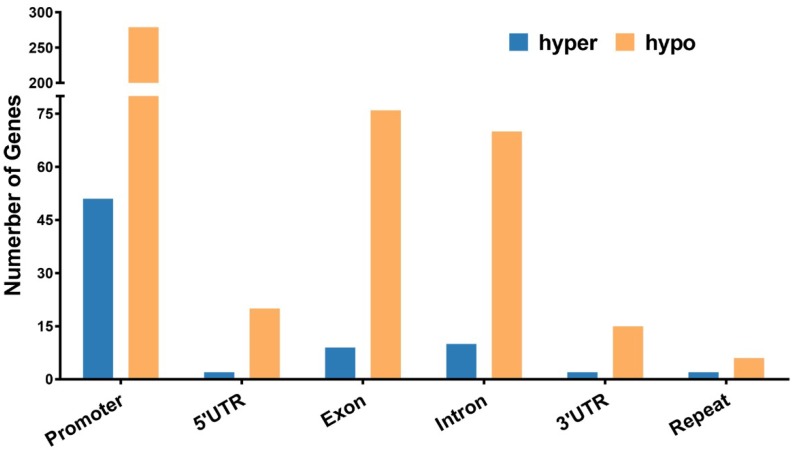
Number of hypomethylated and hypermethylated DMGs in gene functional regions. Hypo: hypomethylated; hyper: hypermethylated.

Genes overlapping with DMRs for at least 1 bp in the functional region were defined as DMR-associated genes (DMGs). In total, we identified 410 DMGs, including 62 hypermethylated genes and 348 hypomethylated genes (Table S1). The number of hypomethylated genes was more than five times that of hypermethylated genes. The DMGs in the promoter were dominant, accounting for over 60% of the total DMGs (Figure 5).

### 2.4. Gene Ontology Ananlysis

**Figure 6:**
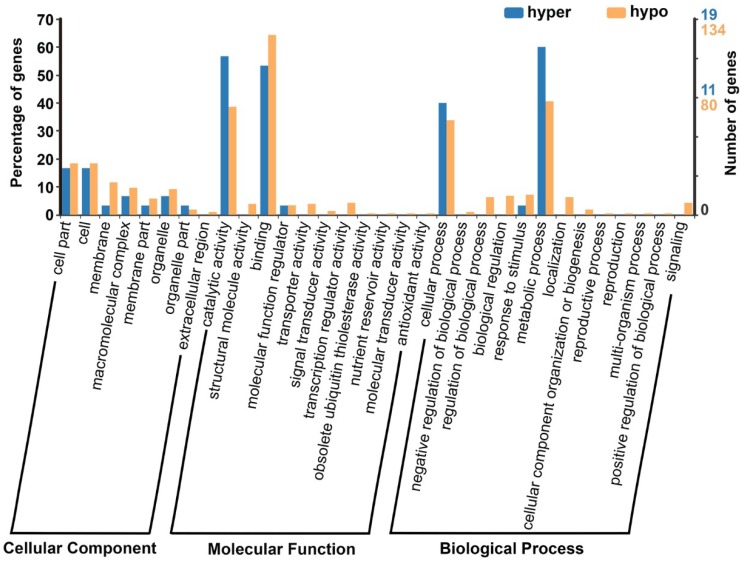
GO analysis of all DMGs. Hypo: hypomethylated; hyper: hypermethylated.

Gene ontology (GO) analysis for the 410 DMGs was performed by using BGI Web Gene Ontology Annotation Plotting (WEGO 2.0, http://wego.genomics.org.cn/) and the results were categorized into three major groups (i.e., biological process, cellular component, and molecular function). The DMGs were annotated to be involved in catalytic activity, binding, cellular process, metabolic process, cell part, organelle, cell and other processes (Figure 6).

### 2.5. DMR-Associated Genes Related to Pollen Development 

Among the 410 DMGs, eleven were identified to have possible functions in flower, anther and/or pollen development. As for the DMGs related to pollen development, three were hypermethylated, including one (*BnaA07g24700D*) in the promoter and two (*BnaA09g28520D* and *BnaCnng55730D*) in the intron; eight were hypomethylated, including four (*BnaA06g05450D*, *BnaA08g08410D*, *BnaC01g33070D* and *BnaC04g12330D*) in the promoter, one (*BnaA03g36790D*) in the intron, one (*BnaA01g35050D*) in the exon, and two (*BnaC08g05510D* and *BnaCnng61870D*) in the spanned regions.

As rapeseed has a close evolutionary relationship with the model plant *Arabidopsis*, all the DMGs were annotated according to their known homologous genes in *A. thaliana*. As a result, there were five DMGs encoding transcription factors, two of which belonged to CCCH zinc finger family: *BnaCnng61870D* was predicted to encode the RING/FYVE/PHD zinc finger superfamily protein and may be involved in pollen development. *BnaA07g24700D*, which is also named as *Callose Defective Microspore1* (*CDM1*), plays a role in regulating callose metabolism around male meiocytes and the integrity of newly formed microspores. *BnaC04g12330D*, which was annotated as *MYB101*, is involved in pollen grain development. *BnaC08g05510D* encodes a pollen-expressed bZIP family transcription factor, and may be involved in regulating male gametophyte development. *BnaA08g08410D* belongs to the HD-ZIP IV family, and is highly expressed in mature pollen with unknown functions.

In addition, *BnaA01g35050D*, which encodes a CLAVATA1-related receptor kinase-like protein, is also known as *Barely Any Meristem 3* (*BAM3*) and is involved in vascular strand development, control of leaf shape, size and symmetry, male gametophyte development and ovule specification. *BnaA03g36790D* is predicted to be a Tesmin/TSO1-like CXC domain-containing protein. *BnaA09g28520D*, a homolog of *TETRAKETIDE a-PYRONE REDUCTASE2* (*TKPR2*), encodes a NAD(P)-binding Rossmann-fold superfamily protein that participates in the biosynthesis of sporopollenin. *BnaC01g33070D* encoding a sterol C-24 reductase cell elongation protein was annotated as *DWARF1*. *BnaCnng55730D*, which is homologous to *GAMMA-TYPE CARBONIC ANHYDRASES 2*, and is involved in anther dehiscence. *BnaA06g05450D* encodes pectinacetylesterase 1 and is considered to be a gene related to tapetum-specific male sterility, but its role in anther development remains unknown. Details for all the DMGs related to pollen development are listed in Table 2.

### 2.6. Validation of the WGBS Data by Traditional Bisulfite Sequencing

**Figure 7:**
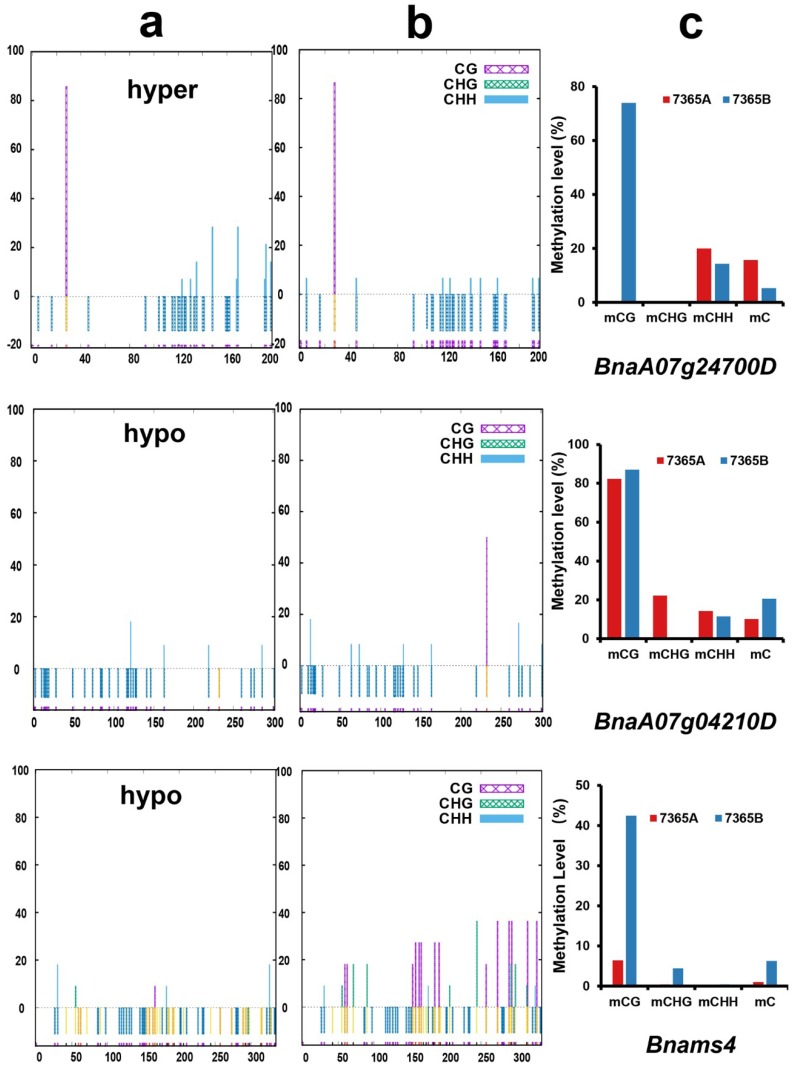
DNA methylation at specific sites of two DMGs and *Bnams4* by traditional bisulfite sequencing. (**a**) Bisulfite PCR results in 7165A; (**b**) Bisulfite PCR results in 7365B. The vertical axis represents the percentage of methylated sites; the horizontal axis represents the position of cytosine in the DMRs; hypo and hyper represents hypo-methylated and hyper-methylated DMR in 7365A. (**c**) WGBS results of the corresponding DMR.

To validate the WGBS data, eight DMGs including five genes related to anther and pollen development were selected to carry out traditional bisulfite sequencing PCR. The bisulfite conversion rates represented by the percentage of unmethylated *BnaIND.a-A3* were over 99% in 7365A and 7365B (Table S3). As shown in Figure 7, 7365A had more methylated cytosines in the DMRs of *BnaA0724700D* and *BnaA09g28520D* and less methylated cytosines in the DMRs of *BnaA07g04210D* and *BnaA08g12350D* (Figure 7, Figures S4-1 and S4-2). *BnaC08g05510D*, *BnaC04g00960D*, *BnaA08g08410D* and *BnaC01g33070D* were hypomethylated in 7365A (Figures S4-1 and S4-2). These results indicated that the traditional bisulfite sequencing results were in good accordance with WGBS data (Table 2, Figure 7, Figures S4-1 and S4-2).

### 2.7. Association between DNA Methylation and Gene Expression

**Figure 8:**
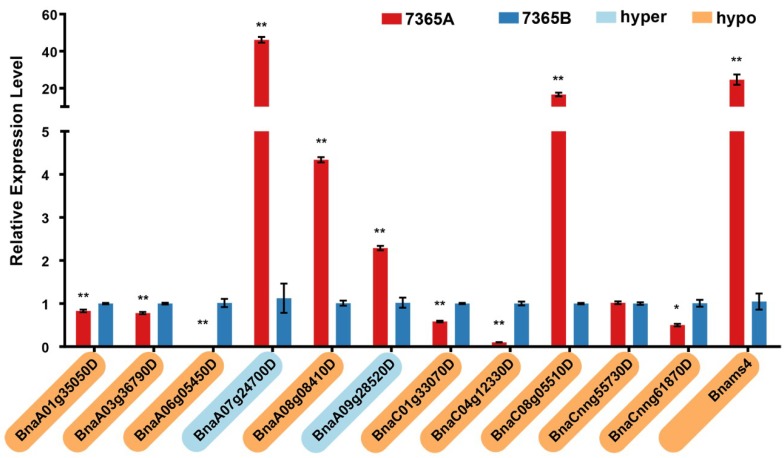
Expression analysis of pollen development-related DMGs and *Bnams4*. The asterisk indicates a significant difference between 7365A and 7365B (*: *p*-value < 0.05, **: *p*-value < 0.01). The Gene ID highlighted in yellow and blue on the vertical axis represents hyper-methylated and hypo-methylated DMRs.

Changes in methylation levels in genomic regions tend to be associated with the differential expression of genes. To analyze the expression of the eleven DMGs associated with pollen development, quantitative reverse transcription PCR (qRT-PCR) was carried out with samples from the young floral buds of 7365A and 7365B. As a result, except for *BnaCnng55730D*, ten other DMGs showed significantly different expression levels between the two lines: among which six (*BnaA01g35050D*, *BnaA03g36790D*, *BnaA06g05450D*, *BnaC01g33070D*, *BnaC04g12330D* and *BnaCnng61870D*) were down-regulated and four (*BnaA07g24700D*, *BnaA08g08410D*, *BnaA09g28520D*, and *BnaC08g05510D)* were up-regulated in 7365A (Figure 8).

### 2.8. Analysis of the DNA Methylation and Gene Expression of Bnams4 

To evaluate the methylation level of *Bnams4,* the causal gene of male sterility in 7365A, the WGBS data were mapped to the insertional fragment where *Bnams4* was located. As shown in the Integrative Genomics Viewer (Figure S5), *Bnams4* was hypo-methylated in 7365A relative to 7365B, especially in the gene body region (Figure S5b). Traditional bisulfite sequencing validated the hypo-methylation in the male sterile line (Figure 7). qRT-PCR results indicated that the expression of *Bnams4* was up-regulated in 7365A (Figure 8).

## 3. Discussion

DNA methylation plays an important regulatory role in plant development and male fertility. Whole genome DNA methylation profiling has been performed in photoperiod/thermo-sensitive male sterile rice [34], CMS soybean [23], and GMS cabbage [21], while no study has described the genome-wide methylation profile in GMS rapeseed. In the present study, global DNA methylation sequencing was for the first time performed on the floral buds of rapeseed GMS line 7365A and its near-isogenic line 7365B.

For all the sequences, the methylation density under the CG context was the highest, followed by that under the CHG and CHH contexts, which is consistent with two other reports on rapeseed [1,35]. The DNA methylation level varies among different tissues and organs. Compared with roots and leaves [1], the average methylation level was lower in young floral buds, especially under the CHH context (Figure S3). Li et al. determined the mean methylation level in cultured microspores under all three contexts [35], which was also lower than that in the roots and leaves. Considering the general role of DNA methylation in silencing genes, the lower methylation level indicated active gene expression in the reproductive organs.

It is worth noting that the mean methylation level of the A_n_ subgenome was significantly lower than that of the C_n_ subgenome under all contexts from both libraries. Chalhoub et al. observed that C_n_ genes presented 4% to 8% higher methylation than their homologous A_n_ genes from the methyl bisulfite sequencing of roots and leaves [1]. A similar result was obtained in cultured microspores by Li et al.: the amount of methylated cytosines distributed on the C_n_ subgenome was obviously larger than that distributed on the A_n_ subgenome [35]. It was hypothesized that the different DNA methylation levels between the A_n_ and C_n_ subgenomes can be attributed to the asymmetrical distribution of TE density in the genome of *B. napus*.

The tendency to variations in DNA methylation across genomic regions was consistent between the male sterile and fertile lines under all methylation contexts. The repeat showed the highest methylation level among the methylated genomic regions, which is in agreement with the role of DNA methylation in silencing repetitive elements to stabilize the genome. The gene body region showed the lowest methylation level, with slightly higher methylation for the intron than for the exon. The methylation level of the promoter greatly increased beyond the TSSs. No significant differences were observed in the comparison of the whole genome cytosines between 7365A and 7365B, indicating that DNA methylation is a relatively stable modification.

We identified a total of 779 DMRs and 410 DMGs, and found that more DMGs were hypomethylated. Recently, a similar tendency was observed in CMS soybean [23]. These results imply that DNA hypomethylation might occur at the initial stage of anther abortion.

There are complex relationships between DNA methylation and gene expression. Generally, DNA methylation in promoters is negatively associated with gene expression. However, we found that *BnaA0724700D* and *BnaA08g08410D* were up-regulated, although they exhibited opposite methylation patterns in their promoters. Except for the up-regulated *BnaA08g08410D*, three other genes (*BnaA06g05450D*, *BnaC01g33070D* and *BnaC04g12330D)* were down-regulated in 7365A, while all the four genes were hypomethylated in their promoters. It is possible that some other factors are involved in the regulation of these genes. Although *BnaCnng55730* showed no differences in expression with hypomethylation in its intron, there could be a positive association between intron methylation and gene expression. *BnaA03g36790D* exhibited down-regulation when hypomethylated. On the contrary, *BnaA09g28520D* was up-regulated with hypermethylation in its intron.

Among the 11 DMGs possibly related to flower, anther and pollen development, several were involved in the pathways similar to those reported by previous studies of 7365A. *BnaA07g24700D*, which was up-regulated in 7365A, plays roles in regulating callose metabolism around male meiocytes and the integrity of newly formed microspores [36]. The synthesis and timely degradation of callose is crucial for the formation of functional microspores. Zhou et al. observed that callose could not be degraded in a timely manner in the *Bnams3* mutant, and identified two β-1,3-glucanase genes (*BnaA6* and *BnaMSR66*) that might be responsible for callose degradation [32]. It is possible that *BnaA07g24700D* plays a role in regulating the two β-1,3-glucanase genes. *BnaA09g28520D* is homologous to *TETRAKETIDE a-PYRONE REDUCTASE2* (*TKPR2*). Coincidentally, in a proteomic comparison between the mutant 7365A and wild-type 7365B, Qin et al. characterized three down-regulated proteins including BnaTKPR1, which forms heterodimer with TKPR2 to participate in sporopollenin biosynthesis in the tapetum [31,37]. *BnaA06g05450D* was predicted to encode pectinacetylesterase 1, which is characterized as a tapetum-specific gene related to male sterility, but its role in anther development remains unclear [38]. Zhu et al. also discovered the unsuccessful separation of tapetum cells and pollen mother cells (PMCs) in 7365A because of the significantly decreased expression of the pectin methylesterase gene [33]. Further research is needed to clarify whether *BnaA06g05450D* also functions in the degradation of specialized cell wall layers surrounding tapetum cells and PMCs. These consistent results indicate the feasibility of applying WGBS to the identification of male sterility-related genes.

In addition, *BnaC04g12330D* was predicted to be MYB101, which functions together with MYB97 and MYB120 in pollen tube–synergid interaction [39]. *BnaC08g05510D* is homologous to a pollen-expressed bZIP transcription factor, which is highly expressed in the tapetum and microspores, but with unknown functions [40,41]. *BnaA08g08410D* was predicted to be the *HD-ZIP IV family-homeodomain GLABROUS 4* (*HDG4*), which is highly expressed in mature pollen without confirmed functions [40]. *BnaA01g35050D* is annotated as *BAM3* in Arabidopsis. BAM1, BAM2 and BAM3 are CLAVATA1-related receptor kinase-like proteins, and the former two are important regulators of early anther development in *Arabidopsis* [42,43]. *BAM3* is possibly involved in male gametophyte development. *BnaA03g36790D* is homologous to *TSO1*, which plays a role in the development of both male and female reproductive tissues, and the *tso1* mutant produced very little pollen [44]. *BnaC01g33070D* is annotated as *DWARF1*, which is involved in the conversion of brassinosteroid precursor 24-methylenecholesterol to campesterol, and the *dwarf1* mutant displayed slightly delayed flowering and reduced fertility [45]. *BnaCnng61870D* was predicted to encode a RING/FYVE/PHD zinc finger superfamily protein, which may participate in pollen development [40]. Further studies are required to investigate the roles of these genes in the male sterility of 7365A.

In the present study, *Bnams4,* the causal gene of male sterility, was found to be hypo-methylated in its gene body region under all contexts and its expression was up-regulated in 7365A. A previous study has shown the hypo-methylation of the upstream region of *Bnams4* under the CHG context, and up-regulated expression in the male sterile line, although the hypo-methylated region is relatively far away (1.4 kb) from the TSS of *Bnams4* [30]. Whether the hypo-methylation of *Bnams4* affects the status of other genes and thus controls male sterility remains to be elucidated.

## 4. Materials and Methods 

### 4.1. Plant Materials, Phenotypic Observation and Sample Collection

The lines 7365A (male sterile; *Bnams3ms3 ms4^b^ms4^b^*) and 7365B (male fertile; *Bnams3ms3 ms4^a^ms4^b^*) were homozygous recessive at the *Bnams3* locus and separated at the *Bnams4* locus [27]. Since they had been cross-pollinated by full sib-mating for more than 10 generations, they were considered as near-isogenic lines (NILs).

The lines 7365A and 7365B were grown in the same research field of Huazhong Agricultural University, Wuhan, China. After anthesis, the male sterile and fertile lines could be easily distinguished from each other according to the significant phenotypic differences in the sizes of their flowers and the fertility of the anthers. The blooming flowers were photographed using a Canon EOS 70D camera. The anthers were separated from floral buds ready to bloom and then gently pressed before staining by acetocarmine, and then photographed using a Nikon 80i optical microscope. Young floral buds 1–2 mm in length (pollen mother cell stage to microspore release from the tetrad) were collected from five plants for each line at the same time. The collected buds were immediately frozen in liquid nitrogen and then stored at –80 °C before DNA and RNA extraction.

### 4.2. Library Construction and Bisulfite Sequencing

Samples were crushed in liquid nitrogen. The total genomic DNA was extracted by Hi-DNA secure Plant Kit (Tiangen Biotech, Beijing, China) according to the manufacturer’s instructions. Genomic DNA degradation and contamination were monitored on agarose gels. DNA purity was checked with the NanoPhotometer^®^ spectrophotometer (Implen, Westlake Village, CA, USA). DNA concentration was measured with Qubit^®^ DNA Assay Kit in Qubit^®^ 2.0 Fluorometer (Life Technologies, Carlsbad, CA, USA).

A total amount of 5.2 μg genomic DNA spiked with 26 ng lambda DNA was fragmented by sonication to 200–300 bp by Covaris S220 (Covaris, Woburn, MA, USA), followed by end repair and adenylation. Cytosine-methylated barcodes were ligated to sonicated DNA according to the manufacturer’s instructions. Then DNA fragments were treated twice using bisulfite with EZ DNA Methylation-GoldTM Kit (Zymo Research, Orange, CA, USA). The resulting single-strand DNA fragments were amplified with KAPA HiFi HotStart Uracil + ReadyMix (2×). Library concentration was quantified by Qubit^®^ 2.0 Fluorometer (Life Technologies, Carlsbad, CA, USA) and quantitative PCR, and the insert size was assayed on Agilent Bioanalyzer 2100 system (Agilent, Santa Clara, CA, USA). The qualified libraries were sequenced on an Illumina Hiseq2500 platform and 125-bp paired-end reads were generated by Novogene Bioinformatics Institute (Beijing, China).

### 4.3. Quality Control

Read sequences produced by the Illumina pipeline in FASTQ format were pre-processed through Trimmomatic (version 0.35) as follows: firstly, as a subset of reads might contain adapter oligonucleotide sequences, the reads containing adapter sequences were filtered out; secondly, since some reads had N (ambiguous bases) in their sequences, any reads with more than 10% N were removed; thirdly, reads with more than 50% low quality bases (PHRED score ≤ 20) were exclude. At the same time, Q20, Q30 and GC content of the data were calculated. The remaining reads that passed all the above filtering steps were counted as clean reads and used for subsequent analyses.

### 4.4. Mapping of the Reads to the Reference Genome

Bismark software (version 0.12.5; Krueger F, 2011) was used for the alignments of bisulfite-treated reads to a reference genome with default parameters. The reference genome was firstly transformed to a bisulfite-converted version (C-to-T and G-to-A conversion) and then indexed by Bowtie2 (version 2.2.5). Sequence reads were also transformed into fully bisulfite-converted versions (C-to-T and G-to-A conversion) before aligning to similarly converted versions of the genome in a directional manner. Sequence reads that produce a unique best alignment from the two alignment processes (original top and bottom strand) were then compared with the normal genomic sequences and the methylation states of all cytosine positions in the read were inferred. Read pairs that shared the same coordinates in the genome were regarded as duplicated, and were removed before methylation state determination to avoid potential calculation bias of the methylation level. The bisulfite non-conversion rate was calculated as the percentage of sequenced cytosines to reference cytosines in the lambda genome.

### 4.5. Differentially Methylated Region (DMR) Analysis

Differentially methylated regions (DMRs) were identified by the swDMR software (https://sourceforge.net/projects/swdmr/) with read coverage ≥ 5, the difference of methylation level ≥ 0.1 or fold change ≥ 2, and with corrected *p*-value < 0.01, using a sliding-window approach. The window was set to 1000 bp and the step length was 100 bp. The Fisher test was implemented to detect the DMRs. Genes overlapping with significant DMRs for at least 1 bp in the functional region were defined as DMR-associated genes (DMGs).

### 4.6. Gene Ontology Analysis of DMR-Associated Genes

Gene ontology (GO) analysis of the DMGs was performed by the GOseq R package, in which gene length bias was corrected. GO terms with corrected *p*-values lower than 0.05 were considered as significantly enriched by DMGs. All the DMGs were annotated by BGI Web Gene Ontology Annotation Plotting (http://wego.genomics.org.cn/).

### 4.7. Data Validation by Traditional Bisulfite Sequencing PCR 

Traditional bisulfite sequencing PCR was used to validate the WGBS data. One microgram genomic DNA was treated for sodium bisulfite conversion according to the protocol of Qiagen EpiTect Bisulfite kit (Cat. No. 59104). The treated DNA was then purified by the Qiagen PCR purification kit (Cat. No. 28106) before being used as bisulfate sequencing PCR (BS-PCR) templates. Primers were designed on Kismeth (http://katahdin.mssm.edu/kismeth/primer_design.pl). BS-PCR was performed with 2× Taq Master Mix (Vazyme Biotech, Nanjing, China). The amplicons were cloned into pMD18-T (Takara Biotechnology, Dalian, China). Then, 10–14 positive clones for each PCR product were sequenced by Sangon Biotech (Shanghai, China) Co. Ltd. The sequencing results were trimmed to remove the vector and primer sequences by the Seqman program of DNAstar software before being analyzed on the Kismeth website [46]. The *BnaIND.a-A3* [47] was used to confirm bisulfite conversion efficiency.

### 4.8. First-Strand cDNA Synthesis and Quantitative Real-Time PCR

Total RNA was extracted from the same plants used for WGBS and purified by RNA prep pure Plant Kit (Tiangen Biotech, Beijing, China). One microgram of total RNA was reverse-transcribed according to the protocol of the Revert Aid First Strand cDNA Synthesis Kit (Thermo Scientific, Waltham, MA, USA), and the cDNA was used as the template for the following amplification. The quantitative RT-PCR (qRT-PCR) was conducted on a CFX96 Realtime system (Bio-Rad, Hercules, CA, USA) using the SYBR Green Real-time PCR Master Mix (Toyobo, Osaka, Japan) with gene-specific primers (Table S4). Each reaction was performed with four replicates by using *BnaActin3* as internal control. The relative level of gene expression was evaluated by the 2^−ΔΔCT^ method [48]. The gene expression levels in 7365B were used to normalize the relative expression. Student’s t-test was adopted to measure the differences in the relative expression of genes between 7365A and 7365B.

## Figures and Tables

**Table 1:** Summary of genome-wide methylation sequencing data.

Sample	Raw Reads	Clean Reads	Uniquely Mapped Reads	Uniquely Mapped Rate (%)	Mean Coverage	Coverage of 5× Depths (%)	Conversion Rate (%)
7365A	228 million	223 million	151 million	67.79	40.15	72.25	99.901
7365B	230 million	225 million	156 million	69.41	41.63	73.09	99.897

**Table 2:** Information of eleven DMR-associated genes involved in pollen development.

Differentially Methylated Gene	Gene Region	Hyper/Hypo	Expression	*p*-Value	Homolog in Arabidopsis	*E*-Value	Predicted Functional Description
*BnaA01g35050D*	exon	Hypo	Down	1.21 × 10^−49^	*AT4G20270*	0	Leucine-rich receptor-like protein kinase family protein (BAM3)
*BnaA03g36790D*	intron	Hypo	Down	0.007581	*AT3G22780*	0	Tesmin/TSO1-like CXC domain-containing protein
*BnaA06g05450D*	promoter	Hypo	Down	3.77 × 10^−52^	*AT1G09550*	2.00 × 10^−143^	Pectinacetylesterase family protein
*BnaA07g24700D*	promoter	Hyper	Up	0.032969	*AT1G68200*	1.00 × 10^−49^	Zinc finger C-x8-C-x5-C-x3-H type family protein (CDM1)
*BnaA08g08410D*	promoter	Hypo	Up	3.14 × 10^−21^	*AT4G17710*	0	homeodomain GLABROUS 4 (HDG4)
*BnaA09g28520D*	intron	Hyper	Up	0.000011	*AT1G68540*	1.00 × 10^−113^	NAD(P)-binding Rossmann-fold superfamily protein (TKPR2)
*BnaC01g33070D*	promoter	Hypo	Down	9.94 × 10^−6^	*AT3G19820*	0	cell elongation protein/DWARF1/DIMINUTO
*BnaC04g12330D*	promoter	Hypo	Down	1.93 × 10^−7^	*AT2G32460*	0	MYB domain protein 101 (MYB101)
*BnaC08g05510D*	exon, intron, utr3	Hypo	Up	6.34 × 10^−23^	*AT1G35490*	6.34 × 10^−23^	bZIP family transcription factor
*BnaCnng55730D*	intron	Hypo	Unchanged	3.58 × 10^−68^	*AT1G47260*	0	Gamma carbonic anhydrase 2
*BnaCnng61870D*	exon, intron, utr5, promoter	Hypo	Down	4.22 × 10^−25^	*AT2G37950*	1.00 × 10^−163^	RING/FYVE/PHD zinc finger superfamily protein

Hyper: hypermethylation; hypo: hypomethylation. down: down-regulated expression in 7365A. up: up-regulated expression.

## Supplementary Materials

Supplementary materials can be found at http://www.mdpi.com/1422-0067/19/9/2689/s1. Figure S1: Circle plot of global DNA methylation level differences between 7365A and 7365B; Figure S2: DNA methylation levels under three contexts in nineteen chromosomes; Figure S3: Different methylation level in roots, leaves and flowers; Figures S4-1 and S4-2: DNA methylation at specific sites of three DMGs by traditional bisulfite sequencing; Figure S5: Integrative Genomics Viewer screen capture of *Bnams4* methylation level. Figure S6: Average density of DNA methylation in different genomic functional regions; Table S1: Information of total methylated genomic region (DMR)-associated genes (DMGs); Table S2: Information of total methylated genomic regions (DMRs); Table S3: Bisulfite conversion efficiency represented by *BnaIND.a-A3* unmethylated rate; Table S4: Primers used in this study. The raw data have been uploaded to NCBI under the accession number SRR7763971 (7365A) and SRR7763972 (7365B).

## Abbreviations

BAM	Barely any meristem
BS-PCR	Bisulfite sequencing polymerase chain reaction
MS	Male sterility
CMS	Cytoplasmic male sterility
GMS	Genic male sterility
CDM 1	Callose defective microspore 1
DMG	Differentially methylated region associated gene
DMR	Differentially methylated region
GO	Gene ontology
HDG4	HD-ZIP IV family-homeodomain GLABROUS 4
NIL	Near-isogenic line
PMC	Pollen mother cell
RdDM	RNA-directed DNA methylation
TE	Transposable element
TKPR2	TETRAKETIDE a-PYRONE REDUCTASE2
TSS	Transcription start site
WGBS	Whole-genome bisulfite sequencing
