# Supplementary material for: Genome-Wide DNA Methylation Comparison between Brassica napus Genic Male Sterile Line and Restorer Line
Source: Int J Mol Sci. 2018 Sep 10;19(9):2689. doi: 10.3390/ijms19092689 (PMC6165103; doi:10.3390/ijms19092689)
Supplement: Supplementary file 1 [file ijms-19-02689-s001.zip › Supplementary material 2.docx]

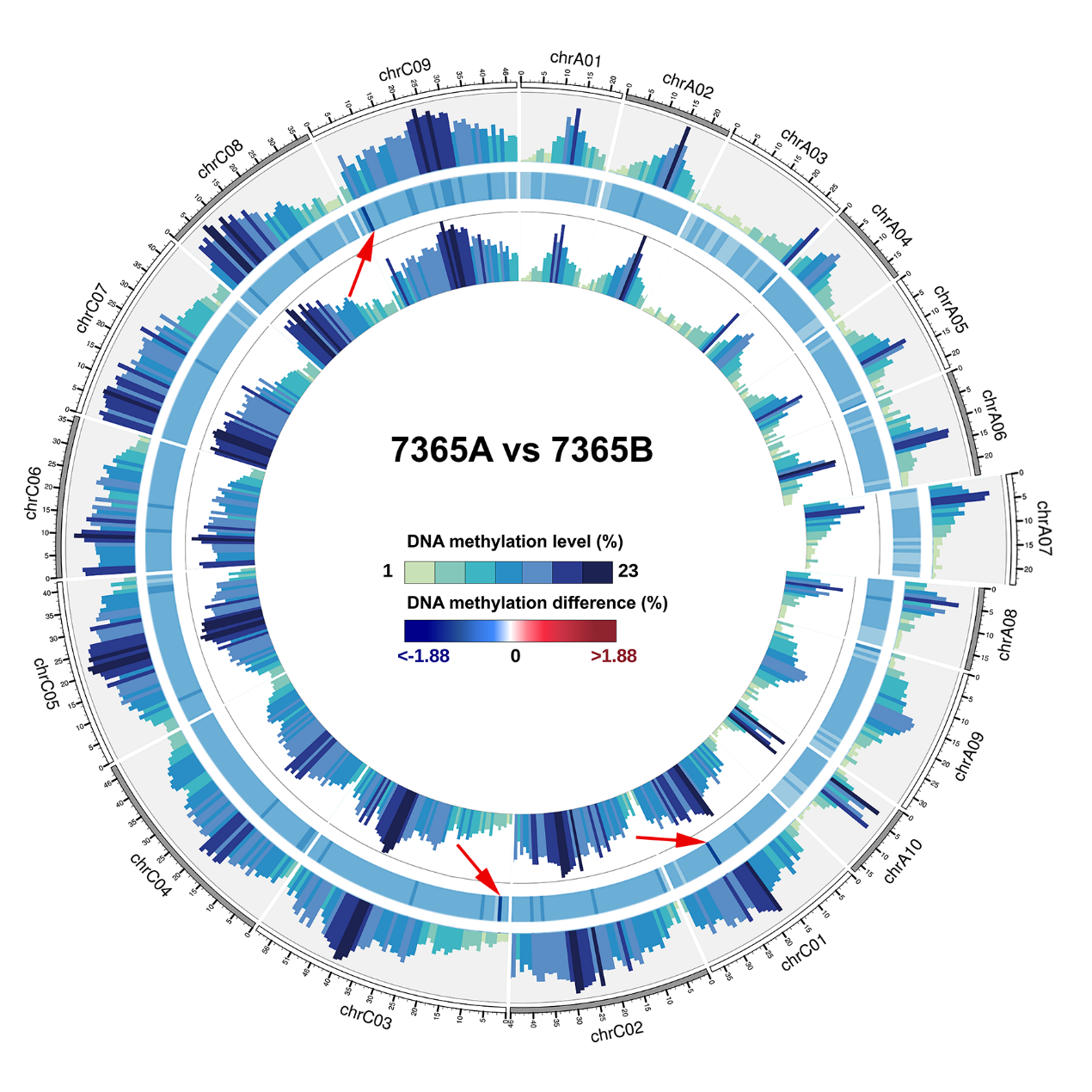
**Figure S1.** Circle plot of global DNA methylation level differences between 7365A and 7365B. The outermost and innermost layers represent the genomic DNA methylation level of 7365A and 7365B, respectively. The middle layer represents the difference in methylation. Red arrows indicate local methylation differences. The chromosome A07 where *Bnams4* locates is highlighted by protruding from the circle.


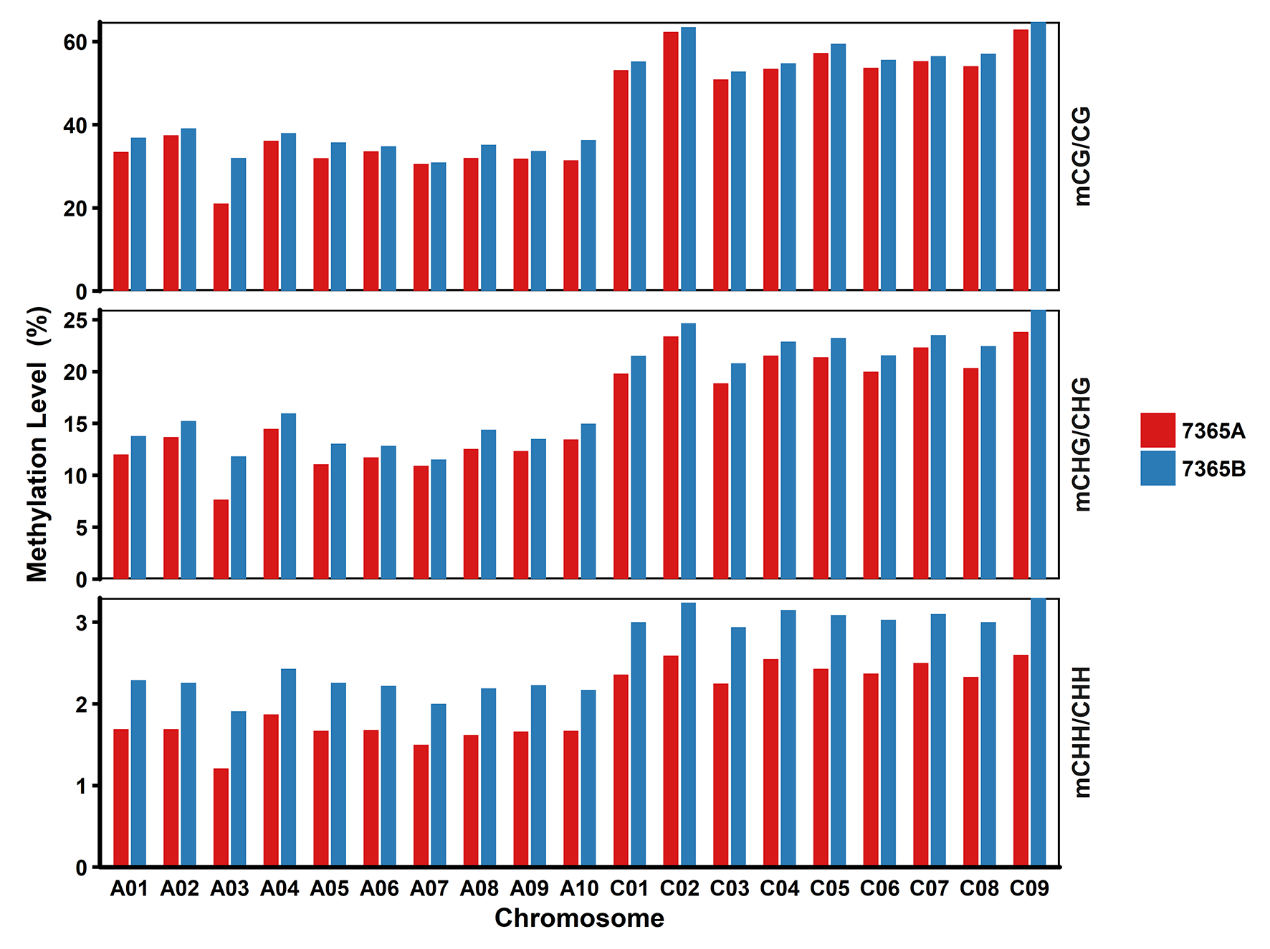
**Figure S2**. DNA methylation levels under three contexts in nineteen chromosomes.


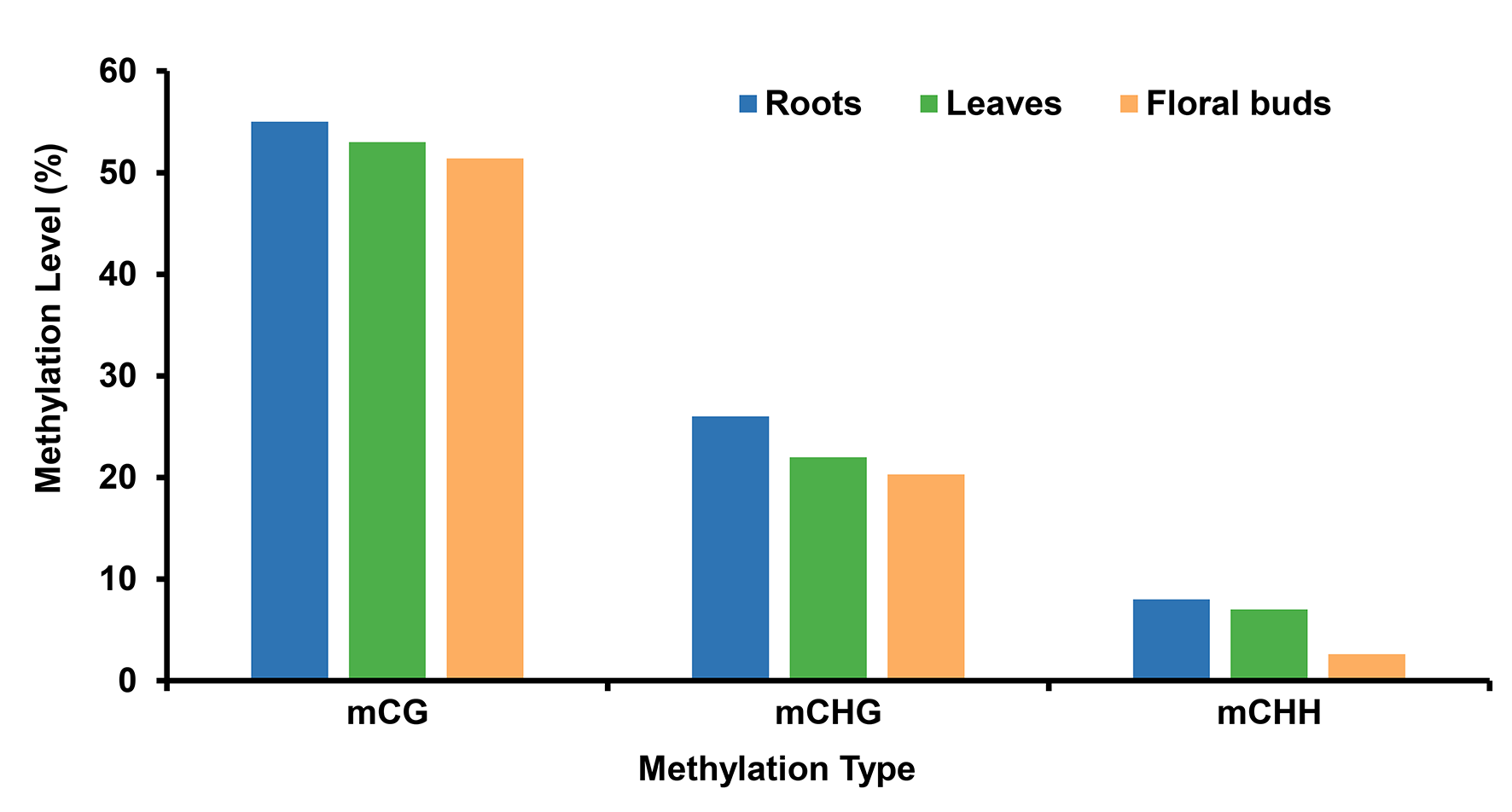
**Figure S3**. Different methylation level in roots, leaves and floral buds. The data of roots and leaves were collected from Chalhoub et al [1].


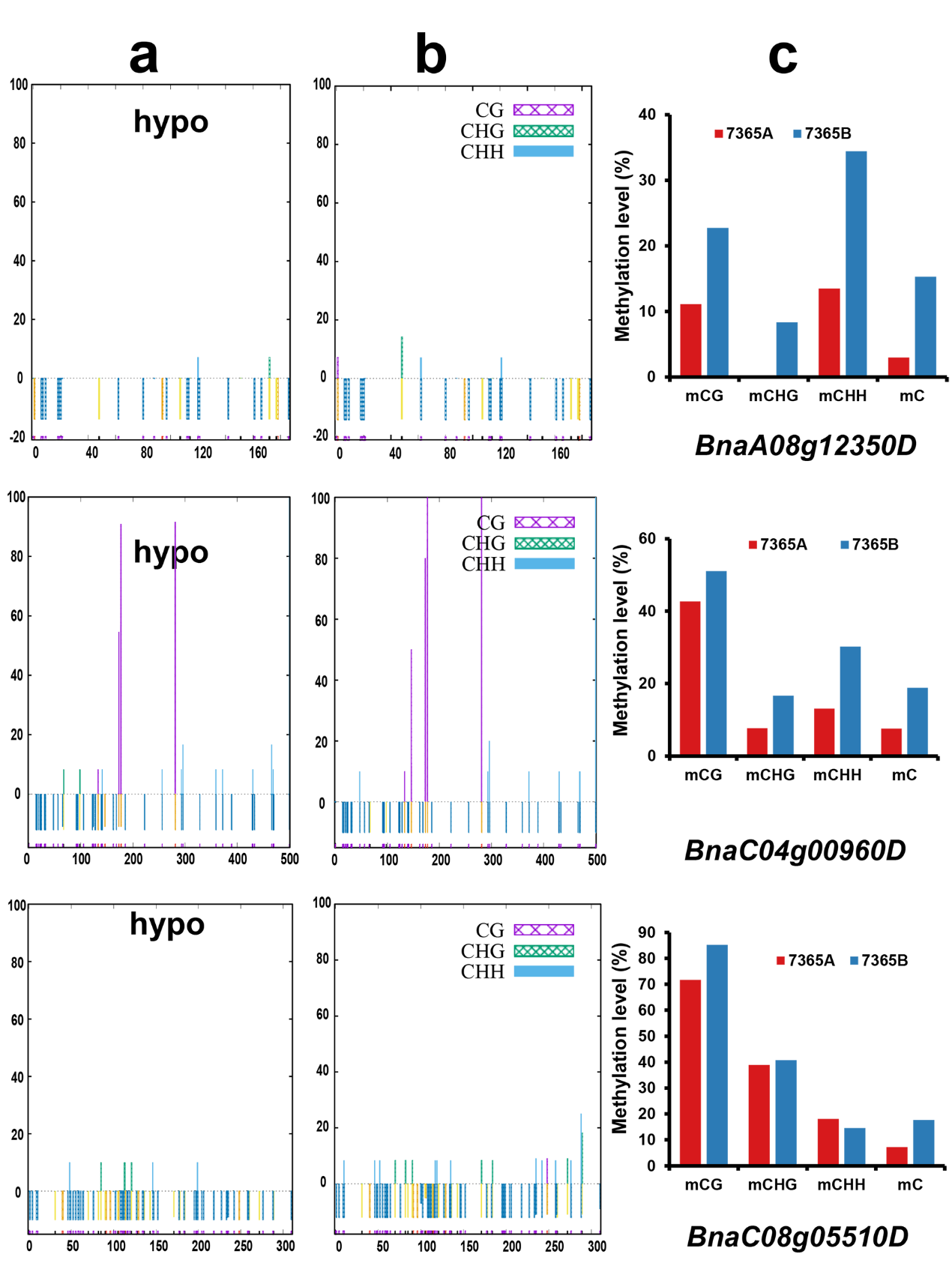
**Figure S4-1**. DNA methylation at specific sites of three DMGs by traditional bisulfite sequencing. (**a**) Bisulfite PCR result in 7365A. (**b**) Bisulfite PCR result in 7365B. The vertical axis represents the percentage of methylated sites; the horizontal axis represents the position of cytosine in the DMRs; hypo and hyper represents hypo-methylated and hyper-methylated DMR in 7365A. **(c)** WGBS result of the corresponding DMR.


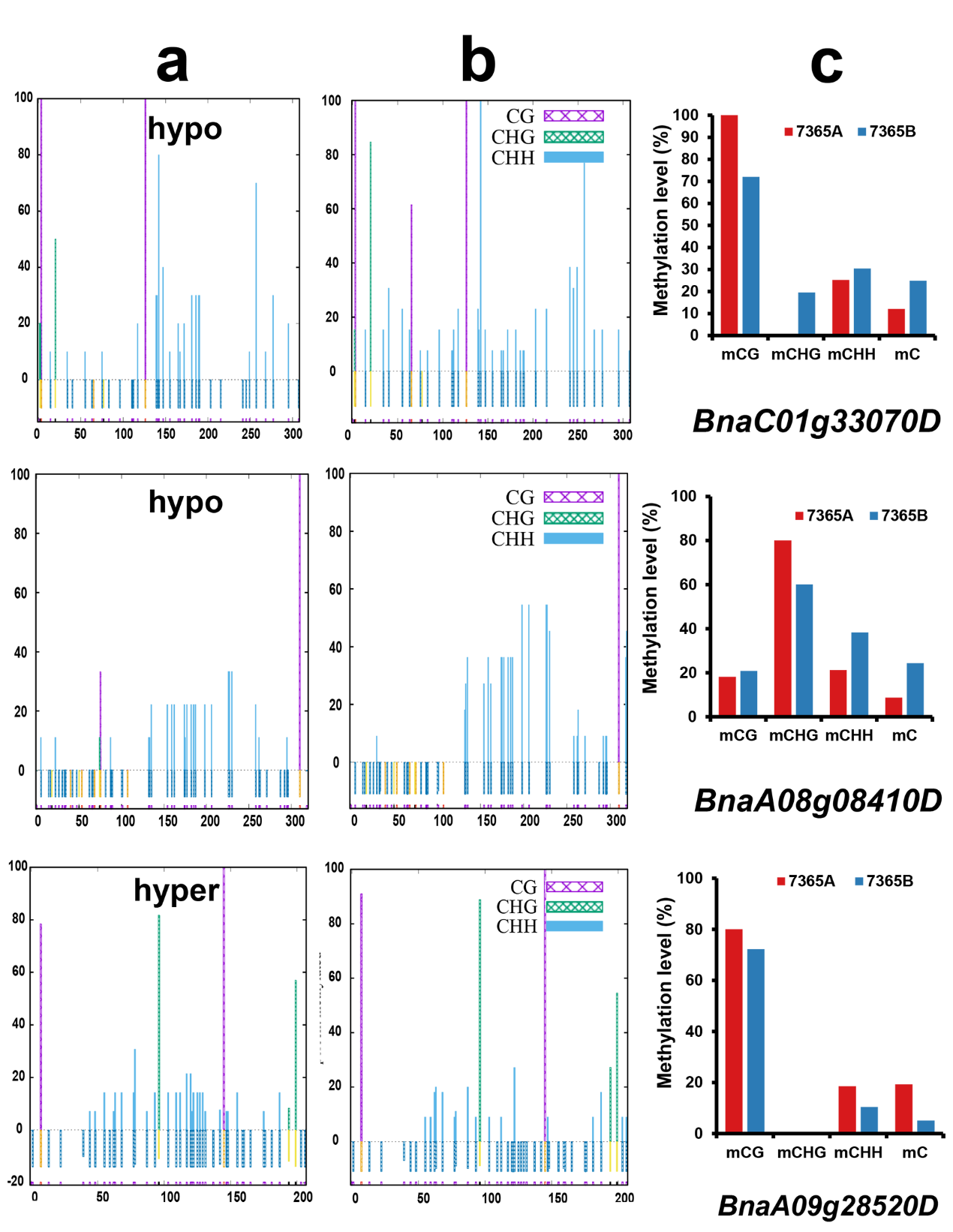
**Figure S4-2**. DNA methylation at specific sites of three DMGs by traditional bisulfite sequencing. (**a**) Bisulfite PCR result in 7365A. (**b**) Bisulfite PCR result in 7365B. The vertical axis represents the percentage of methylated sites; the horizontal axis represents the position of cytosine in the DMRs; hypo and hyper represents hypo-methylated and hyper-methylated DMR in 7365A. (**c**) WGBS result of the corresponding DMR.


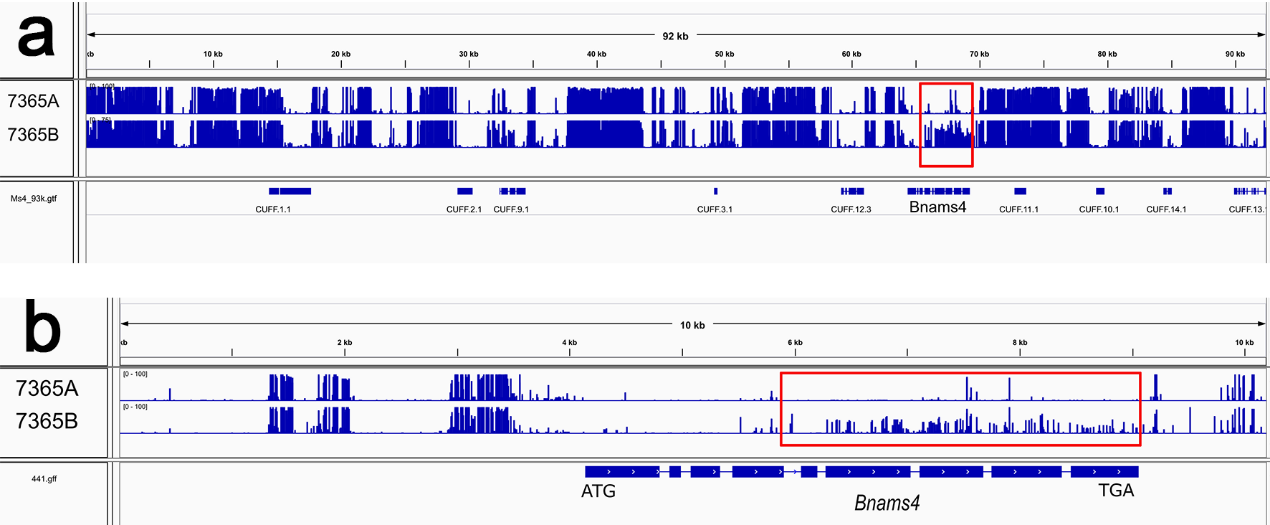
**Figure S5.** Integrative Genomics Viewer screen capture of *Bnams4* methylation level. (**a**) View of insertional region that the *Bnams4 located*. (**b**) View of Bnams4. The height of blue bar in blue represents methylation level. Red boxes indicate methylation difference.


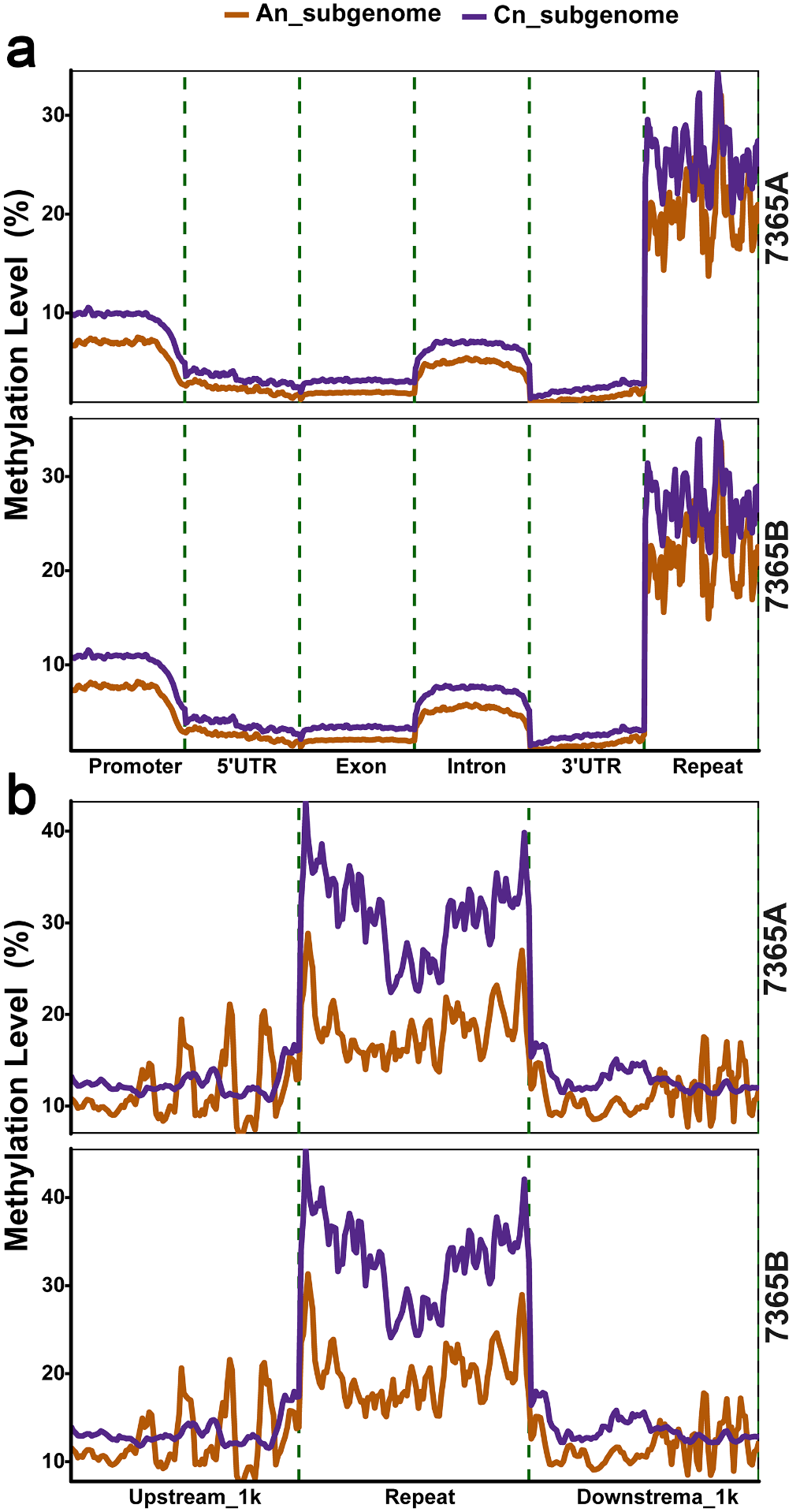


**Figure S6** Average density of DNA methylation in different genomic functional regions. (**a**) Average density of gene methylation on A_n_ and C_n_ subgenome. (**b**) Average density of simple repeat methylation on A_n_ and C_n_ subgenome.

**Table S3**. Bisulfite conversion efficiency represented by *BnaIND.a-A3* unmethylated rate

| **Total Unmethylated rate** | **7365A** | **7365B** |
| --- | --- | --- |
| CG | 99.53% | 100% |
| CHG | 99.22% | 99% |
| CHH | 99.54% | 98.23% |
| All | 99.47% | 99.09% |

**Table S4**. Primers used in this study

| **Primer** | **sequence** |
| --- | --- |
| BS-BnaA07g04210D-F | GTTTTATAYTTTTTTGGAGGTTATG |
| BS-BnaA07g04210D-R | CTACTCTCAAATTTARTAATCCTAT |
| BS-BnaA07g24700D-F | GAATTTTTTTTYGATTTTGTTTTTYGATTT |
| BS-BnaA07g24700D-R | CCACATTTCATRTTTACACTAATCAATTTT |
| BS-BnaA08g08410D-F | AAATYTTTTGGYAGTAGAAAGTAG |
| BS-BnaA08g08410D-R | RTCATATATATACTTTARCCACATT |
| BS-BnaA08g12350D-F | GTAAYATTAGAAGATGAAAATTAGG |
| BS-BnaA08g12350D-R | CATRATCARACCAAAATAAACAAAA |
| BS-BnaA09g28520D-F | TTTTTGAAAAAATTYAGATTTTTYTTTATG |
| BS-BnaA09g28520D-R | RTTCTCCACRTTCTTCTCCAA |
| BS-BnaC01g33070D-F | ATATATAATGTTTATTYYGYGAAAG |
| BS-BnaC01g33070D-R | RTAARCCCTATTACCATATTTAAAA |
| BS-BnaC04g00960D-F | GTGGAAGAGATGTGTTAAGTTTTGG |
| BS-BnaC04g00960D-R | ATTCCCCTRAATTRAAAACCAATTC |
| BS-BnaC08g05510D-F | TTTGGGTGGTTGATAYAGTGGAG |
| BS-BnaC08g05510D-R | RAACTTRAACATCCACATTATAATA |
| BS-Bnams4-F | TTATTGAAAGAATGATGGAGGGAAG |
| BS-Bnams4-R | CAATACCAATCACATCATTCCCAAT |
| BS-BnaIND.a-A3-F | GGAGGAGGAGAGGAAGYAGAAGAA |
| BS-BnaIND.a-A3-R | CCTRRCACCATCCTCTTCAATATCC |
| RT-BnaA01g35050D-F | CCTGTTGAGCTTGGATACATGAAGA |
| RT-BnaA01g35050D-R | GTTGAAGTAAACGAATTGCCCTGAA |
| RT-BnaA03g36790D-F | ATGGAGAGCAAGCAAGAGGAGGA |
| RT-BnaA03g36790D-F | TACGGTGGCAGTGGTGTTAAAGG |
| RT-BnaA06g05450D_F | TCTTGTTCAAGCCGCTTCTG |
| RT-BnaA06g05450D_R | ACCCTGCAAGTGAATGAACC |
| RT-BnaA07g24700D_F | CACCAGCAAGAACCATTCTG |
| RT-BnaA07g24700D_R | TGCCCATATGGTGACAGTTG |
| RT-BnaA08g08410D-F | CTTAGGGTTCTCTGTTGTTCCTGTG |
| RT-BnaA08g08410D-R | GAAAGGTTGAGTGTTGCTGTGGT |
| RT-BnaA09g28520D-F | ATGATTTGAAGGACCGAAAGCCAAG |
| RT-BnaA09g28520D-R | AAGTGCGGTAGTGAAGAAGGGAGAG |
| RT-BnaC01g33070D_F | TCCCCATGAACCTCTCTCTG |
| RT-BnaC01g33070D_R | GTGAGAGCTTCCTTCAATACCG |
| RT-BnaC04g12330D-F | TACAATGAGCCAACAATGGAGAAGG |
| RT-BnaC04g12330D-R | AATAGTGTGGTTGAAGGGAAGTTGT |
| RT-BnaC08g05510D_F | AGGTACCTCTGCCTCGAAATC |
| RT-BnaC08g05510D_R | AGGACCGTCTGGTTTTGTG |
| RT-BnaCnng55730D-F | TTAGGAAGAAATATGCACGCAAGGA |
| RT-BnaCnng55730D-R | CTGGAAGAATCAACTCGGCTGG |
| RT-BnaCnng61870D-F | TAGGAGCGATAGAGAACAGGTGG |
| RT-BnaCnng61870D-R | AGTGGAAGAGCCATGATATGAAGAAG |
| RT-Bnams4-F | CTGGCAAGTTTCGCAGTCTCTA |
| RT-Bnams4-R | GCTTCCCTCCATCATTCTTTCA |
| RT-BnaActin3-F | TCCATCCATCGTCCACAG |
| RT-BnaActin3-R | GCATCATCACAAGCATCCTT |
